# Differential regulation of NAB corepressor genes in Schwann cells

**DOI:** 10.1186/1471-2199-8-117

**Published:** 2007-12-20

**Authors:** Rajini Srinivasan, Sung-Wook Jang, Rebecca M Ward, Shrikesh Sachdev, Toshihiko Ezashi, John Svaren

**Affiliations:** 1 Department of Comparative Biosciences, University of Wisconsin-Madison, Madison, WI, USA; 2 Program in Cellular and Molecular Biology, University of Wisconsin-Madison, Madison, WI, USA; 3 Department of Biochemistry, University of Missouri-Columbia, Columbia, MO, USA; 4 Department of Animal Sciences, University of Missouri-Columbia, Columbia, MO, USA

## Abstract

**Background:**

Myelination of peripheral nerves by Schwann cells requires not only the Egr2/Krox-20 transactivator, but also the NGFI-A/Egr-binding (NAB) corepressors, which modulate activity of Egr2. Previous work has shown that axon-dependent expression of Egr2 is mediated by neuregulin stimulation, and NAB corepressors are co-regulated with Egr2 expression in peripheral nerve development. NAB corepressors have also been implicated in macrophage development, cardiac hypertrophy, prostate carcinogenesis, and feedback regulation involved in hindbrain development.

**Results:**

To test the mechanism of NAB regulation in Schwann cells, transfection assays revealed that both *Nab1 *and *Nab2 *promoters are activated by Egr2 expression. Furthermore, direct binding of Egr2 at these promoters was demonstrated in vivo by chromatin immunoprecipitation analysis of myelinating sciatic nerve, and binding of Egr2 to the *Nab2 *promoter was stimulated by neuregulin in primary Schwann cells. Although Egr2 expression activates the *Nab2 *promoter more highly than *Nab1*, we surprisingly found that only *Nab1 *– but not *Nab2 *– expression levels were reduced in sciatic nerve from Egr2 null mice. Analysis of the *Nab2 *promoter showed that it is also activated by ETS proteins (Ets2 and Etv1/ER81) and is bound by Ets2 in vivo.

**Conclusion:**

Overall, these results indicate that induction of *Nab2 *expression in Schwann cells involves not only Egr2, but also ETS proteins that are activated by neuregulin stimulation. Although *Nab1 *and *Nab2 *play partially redundant roles, regulation of *Nab2 *expression by ETS factors explains several observations regarding regulation of NAB genes. Finally, these data suggest that NAB proteins are not only feedback inhibitors of Egr2, but rather that co-induction of Egr2 and NAB genes is involved in forming an Egr2/NAB complex that is crucial for regulation of gene expression.

## Background

Members of the EGR (early growth response) family of transactivators fulfill critical roles in diverse systems, including nervous system development, bone formation, and fertility. Transcriptional activity of three EGR family members (Egr1/NGFI-A, Egr2/Krox20, and Egr3) is regulated by interaction with the NAB (NGFI-A/EGR binding) family of transcriptional corepressors. The Nab1 protein was first identified by screening a yeast two-hybrid library for proteins that interact with a repressive domain within Egr1[1], and Nab2 was subsequently found to share similar properties [2]. Both Nab1 and Nab2 can bind to a conserved domain in Egr1, Egr2 and Egr3. NAB proteins repress activation of several EGR target promoters [3-5], and the repression mechanism involves interaction with the CHD4 subunit of the Nucleosome Remodeling and Deacetylase (NuRD) complex [6].

The most severe phenotypes caused by loss of EGR function have been found in mice lacking the *Egr2*/*Krox20 *gene (hereafter referred to as *Egr2*). Characterization of these mice revealed three principal defects: 1) defective hindbrain segmentation with the loss of rhombomeres r3 and r5 [7,8], 2) defective bone formation [9], and 3) failure of Schwann cells to myelinate peripheral nerves [10-12]. Target genes regulated by Egr2 in Schwann cells include myelin genes such as Myelin Protein Zero and Myelin-associated glycoprotein [10-15]. As a consequence, most mice with a homozygous *Egr2 *disruption die shortly after birth, although mice that are homozygous for a hypomorphic *Egr2 *allele survive somewhat longer [11].

Because of the myelination defect in peripheral nerves of *Egr2 *knockout mice, several groups have screened human patients with peripheral neuropathies for mutations in the EGR2 gene. Mutations in *EGR2 *have been identified in several patients with myelin disorders, such as Charcot-Marie-Tooth (CMT) disease, Dejerine-Sottas Syndrome, and Congenital Hypomyelinating Neuropathy [16-20]. Most of the neuropathy-associated mutations occur within the zinc fingers of EGR2 and prevent DNA-binding [21]. However, one of the EGR2 mutations associated with a very severe congenital hypomyelinating neuropathy [I268N, [16]] prevents binding of EGR2 to NAB corepressors [21]. The importance of NAB corepressors to the regulation of peripheral nerve myelination by Egr2 was recently confirmed by the demonstration that a double knockout of the *Nab1*/*Nab2 *genes results in a phenotype very similar to that of the *Egr2 *knockout: early lethality and peripheral neuropathy resulting from arrested myelination [22]. Moreover, NAB genes have been implicated in several other physiological processes, including macrophage development, cardiac hypertrophy, prostate carcinogenesis, and feedback regulation involved in hindbrain development [23-26].

As critical regulators of peripheral myelination, it is important to probe the mechanism of NAB regulation. Recent work has shown that *Nab1 *and *Nab2 *are induced by neuregulin signaling [22]. Neuregulin signaling plays an extremely important role in axon-derived signals for Schwann cell myelination [27-35]. Moreover, *Egr2 *and NAB expression appear to be closely linked, as *Nab1 *and *Nab2 *are co-regulated with *Egr2 *after nerve crush injury, and both corepressors are induced by ectopic Egr2 expression in cultured Schwann cells [22,36,37]. However, direct regulation of *Nab1 *and *Nab2 *by Egr2 has not been demonstrated in vivo. The following data demonstrate direct regulation of NAB expression by Egr2 in myelinating sciatic nerve, and indicate that other neuregulin-regulated pathways are specifically involved in inducing *Nab2 *expression.

## Results

### Induction of *Nab *promoters by Egr2

**Figure 1:**
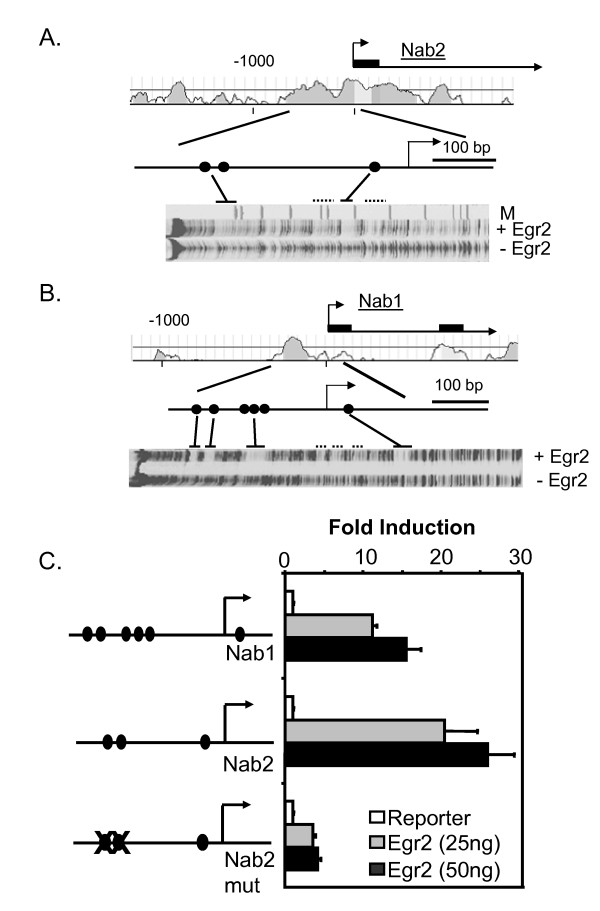
**Egr2 activates the *Nab1 *and *Nab2 *promoters**. A) The plot shows percent identity of the human and mouse *Nab2 *loci upstream of the first exon. DNase I footprinting analysis of a 1000 bp fragment of the mouse *Nab2 *promoter in the presence of recombinant Egr2 protein revealed two footprinted regions (solid line) with some weaker protections (dotted lines). The footprinted regions correspond to three of the four previously identified conserved sites [filled circles, 22] that conform to the Egr2 consensus binding site. There was no apparent footprint over putative site #4 identified in previous sequence analysis of the *Nab2 *promoter [22] M = marker lane. B) A similar analysis of the mouse *Nab1 *promoter confirmed binding of Egr2 to the six previously identified conserved binding sites in the mouse *Nab1 *promoter [filled circles, 22]. C) JEG-3 cells were transfected with a luciferase reporter plasmid containing either the *Nab1 *or *Nab2 *promoter, and the indicated amounts of an Egr2 expression construct. The mutant *Nab2 *promoter contains mutations in the two upstream Egr2 binding sites. Egr2 binding sites are indicated by filled circles. The y-axis shows the fold activation normalized to the activity of the reporter alone. Means and standard deviations of three separate transfections are shown. Activation of both *Nab1 *and *Nab2 *promoters by 25 ng Egr2 (compared to control) was statistically significant (P =< 0.002).

Sequence analysis of the *Nab1 *and *Nab2 *promoters identified several conserved motifs that resemble Egr2 binding sites [22]. To test whether these sites bind Egr2, the mouse *Nab1 *and *Nab2 *promoters were analyzed by DNase I footprinting. In the presence of recombinant Egr2, there were strongly protected regions encompassing three previously identified sites in the *Nab2 *promoter, as well as some weaker protections (Figure 1A). Interestingly, previous analysis of Egr2 site specificity indicated a strong preference for T in the 4^th ^position of the consensus sequence [GCGTGGGCG, [38]], and each of the three strongly protected sites have a T in the 4^th ^base. A similar analysis of the mouse *Nab1 *promoter (Figure 1B) confirmed binding of Egr2 to the conserved binding sites identified previously [22]. The strong binding site at the 3' end of the mouse *Nab2 *promoter corresponds to a site of phorbol ester-induced Egr1 binding in a recent analysis of the human *NAB2 *promoter using electrophoretic mobility shift assays [39].

To test the function of the Egr2 binding sites, fragments encompassing the conserved regions of the *Nab1 *and *Nab2 *promoters were fused to a luciferase reporter gene and transfected into JEG-3 cells, which exhibit a low level of expression of EGR proteins [40]. As shown in Figure 1C, expression of Egr2 activated the *Nab1 *promoter up to 15-fold, and the *Nab2 *reporter was increased 25-fold. Two of the Egr2 binding sites in the *Nab2 *promoter were mutated to determine how that would affect activation by Egr2. As shown in Figure 1, we still observed some residual activation of the promoter, but the induction was much less than observed with the wild type promoter. Previous work had indicated that *Nab2 *is more inducible by serum and neuregulin than *Nab1 *[2,22]. Moreover, the *Nab2 *promoter was more highly induced by Egr2 in the transfection experiments. However, this disparity is somewhat surprising, given that the *Nab1 *promoter contains more Egr2 binding sites than does *Nab2*.

### Neuregulin stimulation increases Egr2 occupancy of the *Nab1 *and *Nab2 *promoters

**Figure 2:**
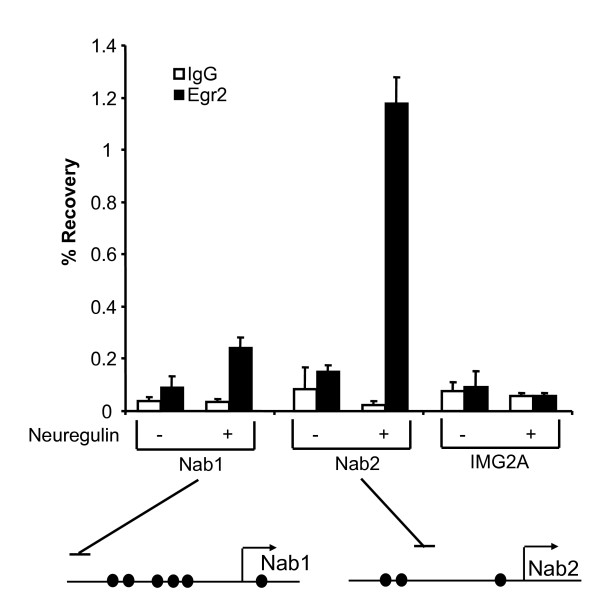
**Neuregulin stimulates Egr2 binding to the *Nab2 *promoter in primary Schwann cells**. Primary rat Schwann cells, untreated or exposed to neuregulin for 2 h, were analyzed by ChIP assay. Formaldehyde-crosslinked chromatin from the two cultures were immunoprecipitated with an antibody for Egr2 (filled bars) or purified control IgG (open bars). Percentage recovery relative to input was determined using quantitative PCR with primers designed for the *Nab1*, *Nab2 *and *Immunoglobin 2A *(*IMG2A*) promoters. The horizontal lines above the promoter diagrams indicate the positions of the amplicons derived from the primer sets used for ChIP analysis. Means and standard deviations were calculated for duplicate measurements from two independent experiments.

**Figure 3:**
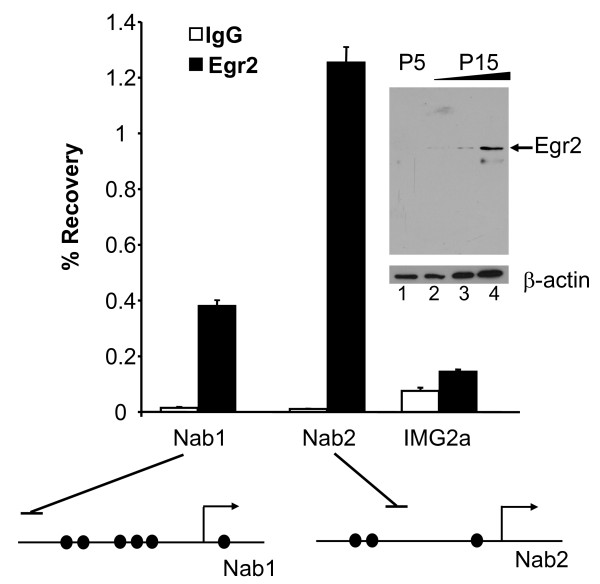
**Egr2 binds NAB promoters in myelinating sciatic nerve**. Formaldehyde-crosslinked chromatin from sciatic nerves of rats at P10 was immunoprecipitated with an antibody for Egr2 (filled bars) or purified control IgG (open bars). Percentage recovery relative to input DNA was determined by quantitative PCR with the same primers used in Figure 2. The horizontal lines above the promoter diagrams indicate the positions of the amplicons derived from the primer sets used for ChIP analysis. Binding at the transcriptionally silent *IMG2A *locus was analyzed as a negative control. The data are representative of at least two independent experiments; average fold enrichment for Egr2 binding (relative to control IgG immunoprecipitation) to the *Nab2 *promoter in four independent experiments was 51-fold (P = 0.016). Inset: Immunoblot analysis with the same antibody used in the ChIP assay was used to detect Egr2 (~70 kD) in rat sciatic nerve lysates at P5 and P15. Three amounts of the P15 lysate (in increasing 2-fold amounts in lanes 2–4) were loaded to facilitate comparison with the expression level at P5. The blot was reprobed with a β-actin antibody as a normalization control (bottom panel).

In rat Schwann cells treated with neuregulin, there is a large induction of Egr2 (within 1 hour), followed by a delayed induction of *Nab1 *and *Nab2*, which peaks at two hours after neuregulin addition [22], with *Nab2 *being more highly induced than *Nab1*. To test if neuregulin stimulates direct binding of Egr2 to the NAB promoters, chromatin immunoprecipitation (ChIP) assays were used, in which formaldehyde is used to covalently crosslink DNA with any associated proteins and the proteins of interest are subsequently immunoprecipitated [14,41]. Primary rat Schwann cells were treated for 2 hours with neuregulin and then cross-linked with 1% formaldehyde. Sonicated chromatin was then immunoprecipitated with an Egr2 antibody or control IgG. An untreated culture of Schwann cells was also cross-linked and immunoprecipitated similarly. After crosslinks were reversed, purified DNA was analyzed by quantitative PCR using primer sets proximal to the Egr2 binding sites in the *Nab1 *and *Nab2 *promoters. As shown in Figure 2, binding of Egr2 to the *Nab1 *and *Nab2 *promoters was significantly stimulated upon treatment with neuregulin. Egr2 binding was not detected at an immunoglobulin promoter [IMG2A, which lacks Egr2 binding sites and is transcriptionally silent in Schwann cells, [14,41]] in either sample. Evidence for the specificity of the antibody used for ChIP analysis includes induction of Egr2 binding by neuregulin (Figure 2), and immunoblot analysis using this antibody reveals a band of the correct size (Figure 3). In addition, this antibody has been extensively tested with multiple negative control sites [6,14,41], and expression of dominant negative Egr2 (consisting of the DNA-binding domain of Egr2) reduces Egr2 binding in this assay (unpublished data). In parallel cultures, *Nab2 *mRNA was induced 11-fold upon treatment with neuregulin for 2 hours, consistent with previously published results [22].

### Egr2 binds to the *Nab1 *and *Nab2 *promoters in myelinating sciatic nerve

We have recently employed ChIP analysis of myelinating sciatic nerve to test whether Egr2 directly regulates specific elements in vivo [6,14,41]. Egr2 expression increases concurrently with initiation of myelination in rat sciatic nerve within two weeks after birth [42], which is also observed in immunoblot analysis of Egr2 expression in P5 and P15 rat sciatic nerve (Figure 3). Therefore, freshly dissected sciatic nerves from rat pups at P10 (from pools of 5–13 pups) were minced in 1% formaldehyde after dissection. After immunoprecipitation of sonicated chromatin, quantitation of the ChIP assays demonstrated significant enrichment of Egr2 at both *Nab1 *and *Nab2 *promoters in rat sciatic nerve (Figure 3).

### *Nab *expression levels in peripheral nerve of Egr2-deficient mice

**Figure 4:**
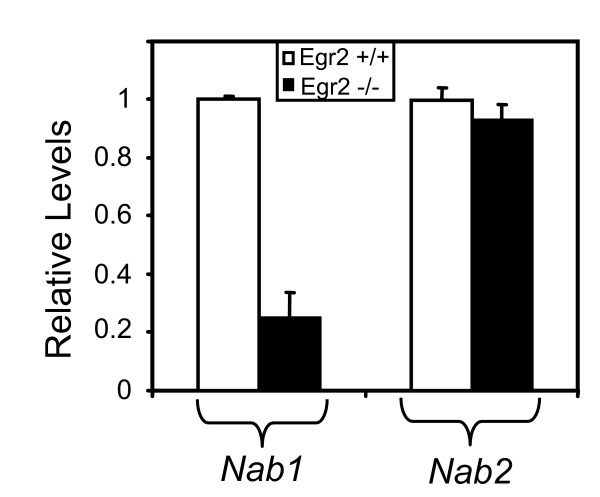
**Expression levels of *Nab *genes in Egr2 null mice**. Sciatic nerves were isolated from *Egr2 *wild type (+/+) and knockout (-/-) mice. The relative levels of the *Nab1 *and *Nab2 *genes are indicated relative to the wild type sample, which was set as 1. Each level was determined from >10 pooled mice of the same genotype and normalized to the level of 18S rRNA. Quantitative PCR assays were performed in duplicate, and the standard deviation is indicated. Further analysis of nerves from individual mice at P7 (3 of each genotype) confirmed reduction of *Nab1 *(expression level of 0.58 for Egr2 -/- relative to 1 for wild type, P = 0.04). Expression level of *Nab2 *in the same samples was 0.95 relative to wild type.

To independently test the regulation of NAB genes by Egr2 in vivo, we examined NAB expression levels in the *Egr2*/*Krox20 *knockout (Figure 4). In these mice, Schwann cells develop normally and associate with axons, but fail to initiate myelination of peripheral nerves [10]. The sciatic nerves of seventeen wild type and knockout animals at P7 were pooled, and the levels of *Nab1 *and *Nab2 *were determined by quantitative PCR. Previous analysis of these samples had indicated that expression of many myelin-associated Egr2 target genes are significantly reduced in the knockout nerves [14,41,43]. Consistent with the data from the transfection and ChIP assays, we observed a significant decrease in the expression of *Nab1*. A similar reduction in *Nab1 *expression was reported in mice with a hypomorphic allele of *Egr2 *[22]. In contrast, the expression level of *Nab2 *was not significantly different in mice containing the null allele of *Egr2*/*Krox20*.

### Regulation of the *Nab2 *promoter by ETS proteins

**Figure 5:**
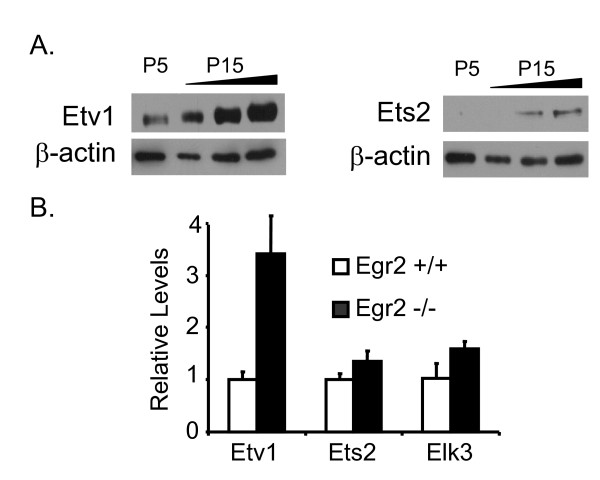
**ETS expression in peripheral nerve**. A) Immunoblot analysis was used to determine protein levels of Etv1 and Ets2 in rat sciatic nerve at P5 and P15. Three amounts of the P15 lysate (in increasing 2-fold amounts) were loaded to facilitate comparison with the expression level at P5. For each antibody, the blot was reprobed with a β-actin antibody as a normalization control. B) Expression levels of *Ets2*, *Etv1*, and *Elk3*, in wild-type (+/+) and homozygous null (-/-) Egr2 mice at P7, were determined by quantitative RT-PCR. The relative levels of each gene were normalized to 18S rRNA and fold induction was determined as compared to wild-type sample, which was set as 1.

**Figure 6:**
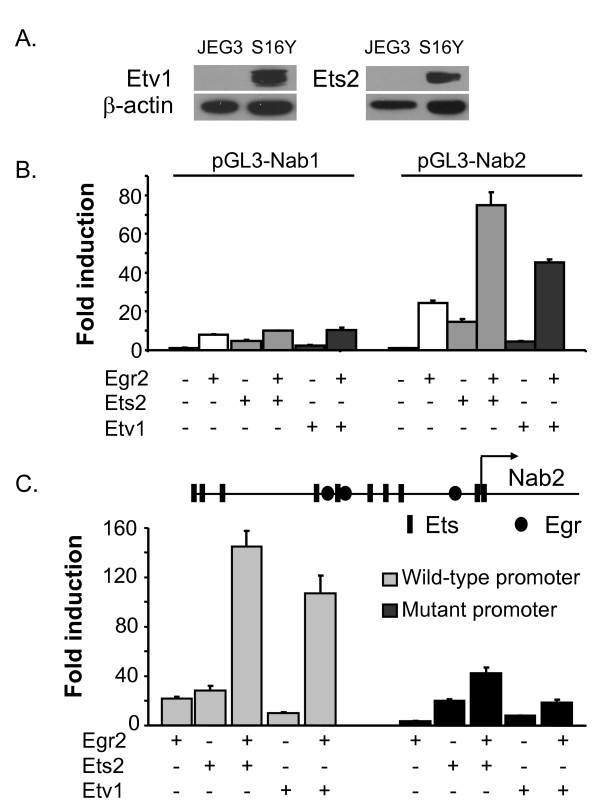
**ETS activation of NAB promoters**. A) Lysates of the JEG3 and S16Y (Schwann cell) lines were probed with the antibodies directed against Etv1 and Ets2, and respective blots were re-probed with β-actin antibody as a loading control. B) JEG3 cells were transfected with either the pGL3-Nab2 or pGL3-Nab1 reporter. Ets2 (25 ng), Etv1 (50 ng), or Egr2 (25 ng) expression plasmids were co-transfected as indicated in each panel. The y-axis shows the fold activation normalized to the activity of the reporter alone. Means and standard deviations of three separate transfections are shown. Average fold induction of the NAB2 promoter by Ets2 and Etv1 in 8 independent transfections was 19.5- and 7.3-fold, respectively (P =< 10^-6 ^for both). C) Similar transfections were performed to compare ETS activation of the pGL3-Nab2 (wild type) promoter with the mutant *Nab2 *reporter construct in which two Egr2 binding sites are mutated (used in Figure 1c). The diagram indicates potential ETS binding sites identified by sequence analysis relative to the footprinted Egr2 binding sites in the *Nab2 *promoter.

Given the unexpected finding that the expression level of *Nab2 *was not lower in the absence of Egr2, the *Nab2 *promoter was screened for other transcription factor binding sites that could contribute to *Nab2 *expression, thereby compensating for the loss of Egr2 activation. Analysis of the *Nab2 *promoter revealed several putative binding sites for ETS transcription factors (diagrammed in Figure 6B). Interestingly, ETS proteins are regulated by neuregulin signaling [44-46]. In addition, previous studies have identified ETS transcription factors as playing an important role in autocrine and neuregulin-mediated survival of Schwann cells, and showed that several ETS genes, including Net/Elk3, Ets2, GABPα and Etv1/ER81, are expressed in E12 and newborn mouse peripheral nerve [47]. Ets2 and Etv1/ER81 (hereafter referred to as Etv1) were selected for further analysis as they were highly expressed in microarray analyses of mouse sciatic nerve [see 94246, 92927 probe sets in supplementary data of [48,49]]. Quantitative PCR analysis confirmed significant levels of *Ets2 *and *Etv1 *in mouse and rat sciatic nerves during peripheral nerve myelination (data not shown), and Western analysis (Figure 5A) showed that both Ets2 and Etv1 are developmentally increased in rat sciatic nerve at P15, compared to the P5 (early myelination) timepoint. Since *Nab2 *expression was unaffected in the *Egr2 *knockout, we assessed the expression levels of *Ets2*, *Etv1*, and *Elk3 *in *Egr2 *knockout nerves from P7 mice. Interestingly, *Ets1 *expression was previously shown to be elevated in sciatic nerve of Egr2-deficient mice [11], and *Etv1 *expression was somewhat higher in the *Egr2 *null mice as compared to wild-type mice (Figure 5B). The presence of Ets2 and Etv1 in mouse and rat sciatic nerve during myelination [47-49], as well as in Egr2 null mice, suggest that ETS transcription factors could compensate in the absence of Egr2 to maintain *Nab2 *expression levels.

### ETS proteins activate the *Nab2 *promoter

To test whether ETS proteins activate the *Nab1 *and *Nab2 *promoters, JEG3 cells were transfected with the *Nab1 *or *Nab2 *reporters described above, along with expression vectors for Ets2 and Etv1. Western analysis revealed that Ets2 and Etv1 expression in JEG3 cells is much lower than found in the S16Y Schwann cell line (Figure 6A). The *Nab2 *promoter was significantly induced by Ets2 and Etv1 expression, and this induction was further potentiated by coexpression of Egr2 (Figure 6B). A dominant-negative ETS construct [consisting of the DNA-binding domain of Ets1, as used previously, [47]] specifically interfered with ETS-mediated induction of the promoter and had no effect on activation by Egr2 (data not shown). In contrast, the *Nab1 *promoter was activated to a lower extent by Ets2 and Etv1, and the ETS factors did not appear to augment activation of the *Nab1 *promoter by Egr2.

We tested the ability of Ets2 and Etv1 to induce a mutated version of the *Nab2 *promoter construct containing mutations in two of the Egr2 binding sites (Figure 6C). As observed previously, the mutant promoter is only weakly induced by Egr2. However, Ets2 and Etv1 were nonetheless able to induce this mutant reporter in the absence of Egr2, although the synergistic activation observed with Egr2 was clearly diminished in the mutant promoter.

### Ets2 binds to the *Nab2 *promoter in S16Y Schwann cells

**Figure 7:**
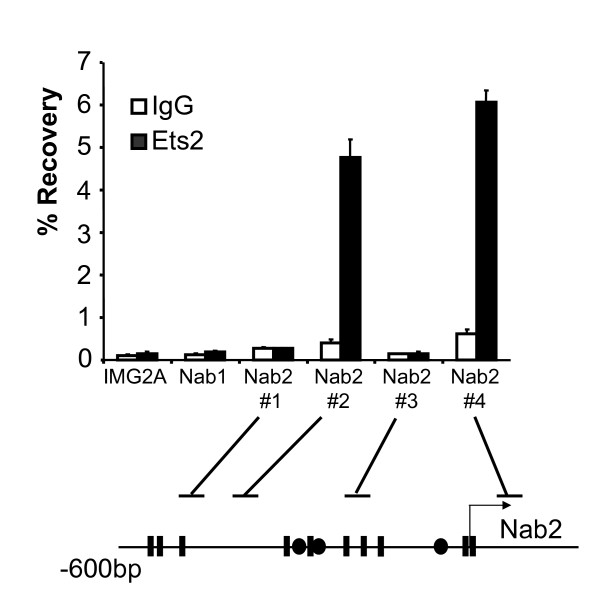
**Ets2 binds to the endogenous *Nab2 *promoter**. Cross-linked chromatin from the S16Y Schwann cell line was immunoprecipitated with Ets2 antibody (filled bars) or purified control IgG (open bars). Percentage recovery relative to input DNA was determined for *IMG2A*, *Nab1*, and several *Nab2 *primer sets as indicated in the diagram. Potential ETS binding sites identified by sequence analysis are shown as filled rectangles. Primer set 3 was used in previous figures to detect Egr2 binding.

In order to test if ETS factors directly regulate *Nab2 *in Schwann cells, we carried out ChIP assays in the S16Y Schwann cell line [50], which expresses significant levels of *Nab1*, *Nab2 *(data not shown) and ETS factors (Figure 5A). S16Y cells were crosslinked in 1% formaldehyde and sonicated chromatin was immunoprecipitated with either an affinity purified Ets2 antibody (used previously for ChIP analysis, [51]) or an IgG control antibody. Quantitative PCR was carried out on purified DNA with primer sets in the *Nab1 *and *Nab2 *promoters, and the *IMG2A *locus as a negative control. As shown in Figure 7, we observed significant enrichment of Ets2 using two primer sets in the *Nab2 *promoter, whereas binding of Ets2 was not detected at either the *Nab1 *promoter or the *IMG2A *negative control locus. A similar trend in Ets2 occupancy was observed in the S16 Schwann cell line ([50], data not shown).

One limitation of the ChIP assay is that it cannot be used to precisely localize binding of transcription factors. However, the analysis suggests that Ets2 binding is separable from Egr2, because Ets2 binding was not detected using the primer set (#3) that was used previously to detect binding of Egr2 within the *Nab2 *promoter (Figures 2 and 3). In addition, the relatively simple recognition site of ETS factors (GGAA or GGAT) does not make sequence analysis necessarily predictive, since the upstream cluster of putative ETS binding sites (spanned by primer set 1) does not appear to bind Ets2. Accordingly, deletion of the upstream cluster of putative ETS binding sites does not affect activation of the *Nab2 *promoter in transfection assays (data not shown). Ets2 binding appears to occur in two separable regions of the *Nab2 *promoter, as detected by primer sets 2 and 4. Interestingly, primer set 4 overlaps with the corresponding segment of the human NAB2 promoter that was recently shown to be inducible by phorbol ester [39], which also activates ETS proteins. Overall, these data are consistent with regulation of the *Nab2 *promoter by ETS transactivators in vivo.

## Discussion

Recent studies have shown that NAB proteins are required for peripheral nerve myelination by Schwann cells, as mice lacking *Nab1*/*Nab2 *displayed a severe congenital hypomyelinating phenotype similar to *Egr2 *knockout mice [22]. The molecular roles of Egr2 and NAB proteins appear to be closely intertwined at several levels, including both physical interaction and coregulation [2,21-24]. Several previous studies have suggested that EGR factors regulate the expression of Nab1 and Nab2 corepressors. First, *Nab2 *and, to a lesser extent, *Nab1 *are activated in a delayed early fashion by a variety of stimuli that induce expression of EGR family members [2,23,39,52,53]. For example, neuregulin treatment of Schwann cells stimulates *Egr2 *and activates *Nab1 *and *Nab2 *expression in a slightly delayed manner [22]. Second, the *Nab2 *promoter can be activated by EGR factors in transient transfection studies [[39], Figure 1, [54]], and ectopic expression of Egr1 and Egr2 stimulates expression of endogenous *Nab1 *and *Nab2 *in a variety of cell lines, including Schwann cells [22,36,37,55,56].

These earlier studies, however, did not test direct regulation of NAB corepressors by Egr2. Our data provide several lines of evidence to establish *Nab2 *regulation by Egr2 in vivo. First, DNAse footprinting analysis identified several Egr2 protected sites in both *Nab1 *and *Nab2 *promoters. Second, activation of *Nab2 *by Egr2 in transient transfection assays was severely compromised when Egr2 binding sites were mutated (Figure 1). Finally, ChIP assays demonstrated direct binding of Egr2 to both promoters in cultured Schwann cells as well as myelinating sciatic nerve (Figures 2 and 3). Several studies have implicated neuregulin signaling as a critical factor in Schwann cell survival and stimulation of myelination [27-34]. Egr2 occupancy at both *Nab1 *and *Nab2 *was significantly enhanced upon neuregulin stimulation of Schwann cells. Our results provide the first demonstration of neuregulin-induced binding of a transcription factor to a specific promoter in Schwann cells.

Several groups have shown that Type III neuregulin (membrane-bound) plays a central role in regulating myelination in vivo [29,30,35]. Therefore, addition of soluble neuregulin (as in Figure 2) does not act as a mimic of myelination. Nonetheless, Egr2 is regulated by both soluble neuregulin and interaction with Type III neuregulin [22,57,58], and therefore soluble neuregulin probably induces some of the early pathways relating to Egr2 induction in vivo. Since Egr2 expression is regulated by Type III neuregulin signaling in vivo [58], our ChIP data in myelinating rat sciatic nerve presumably reflect type III neuregulin-dependent binding of Egr2 to the NAB promoters.

Previous work showed that expression of *Nab1 *and *Nab2 *in zebrafish hindbrain is dependent on *Egr2 *expression [26]. Therefore, it was surprising that *Nab2 *levels were not affected in *Egr2 *knockout mice. In contrast, *Nab1 *levels were significantly lower in the absence of Egr2, even though the *Nab1 *promoter is less effectively activated by neuregulin and *Egr2 *expression (Figure 1, [22]). These data suggested that *Nab2 *might be coregulated by other factors that compensate for loss of Egr2, and promoter analysis revealed several conserved binding sites for ETS transcription factors. ETS family members are activated by neuregulin signaling [45-47]. Since *Nab2 *is more highly induced by neuregulin stimulation [22], we tested whether ETS proteins are involved in regulation of *Nab2*.

Both Ets2 and Etv1 activated the *Nab2 *promoter in transfection assays, and we observed a cooperative effect on the *Nab2 *promoter when Egr2 was co-expressed together with Ets2 or Etv1. Furthermore, chromatin immunoprecipitation analysis with an Ets2 antibody detected binding in the *Nab2 *promoter, but not in the *Nab1 *promoter. Unfortunately, we have been unable to find an Etv1 antibody that is suitable for ChIP analysis, but recent immunohistochemical studies indicate that Etv1/ER81 is expressed in Schwann cells [59]. Based on our data, we propose that the higher induction of *Nab2 *by neuregulin, compared to *Nab1 *[22], is due to synergistic activation of the *Nab2 *promoter by Egr2 and ETS factors. Consequently, the maintained expression of Nab2 levels in the knockout could be mediated by elevated levels of Etv1 (Figure 5), Ets1 [11], or other ETS factors that may be revealed by a more comprehensive analysis of family member expression. In addition, although *Ets2 *mRNA is not induced in the *Egr2 *knockout, its activity (as well as that of other ETS factors) could be elevated post-transcriptionally (e.g. phosphorylation).

In contrast, the *Nab1 *promoter was more modestly activated by ETS proteins, suggesting that *Nab1 *is more exclusively dependent on EGR factors and consequently is more reduced in the absence of Egr2. However, the modest induction of the *Nab1 *promoter by ETS factors in transfection assays could reflect some level of regulation of *Nab1 *expression by ETS proteins in Schwann cells, particularly if there are other ETS binding sites that lie outside of the *Nab1 *promoter regions we have analyzed. A limitation of these studies is that the importance of the NAB promoters relative to more remote enhancer elements has not been established. The mechanism of Nab2 activation by ETS remains to be determined, since only potential ETS binding sites in the *Nab2 *promoter have been identified. Despite the commonality of the core ETS motif (GGAa/t), individual ETS factors have distinct binding site preferences. Therefore, future studies will focus on identifying the relevant ETS factor(s) using RNAi approaches and/or knockout mice in order to facilitate such a mechanistic analysis.

Given the in vivo evidence that ETS proteins regulate expression of *Egr1 *and *Egr2 *[23,60-62], it seems likely that co-induction of *Egr2 *and *Nab2 *by ETS transcription factor activation may have evolved to ensure formation of sufficient Egr2/NAB complexes that are required for specific gene regulation events in Schwann cell differentiation. This may also prevent inappropriate activation of some target genes if Nab2 was not immediately present at the onset of Egr2 induction. Alternatively, it is possible that Nab2 modulates other transcription factors, so that its induction should not exclusively depend on Egr2.

**Figure 8:**
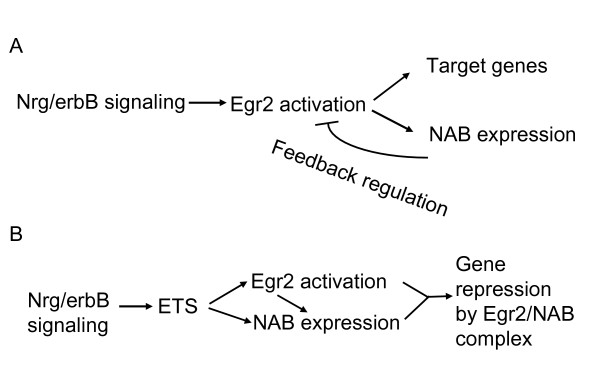
**Model for regulation of NAB expression**. The figure indicates a model for regulation of NAB proteins during myelination, based on the requirement of NAB proteins for peripheral nerve myelination. In the first model (A), neuregulin/erbB signaling (and/or other axon-dependent signals) trigger expression of *Egr2/Krox20 *in myelinating Schwann cells, and the induction of NAB proteins by binding of Egr2/Krox20 acts as a negative feedback control to limit activation of specific target genes that are important for myelination. B) The second model suggests that co-induction of Egr2 and NAB proteins is mediated by neuregulin-activated expression of ETS factors. Activation of ETS factors induces sufficient amounts of NAB proteins so that an Egr2/NAB complex can actively repress specific target genes. It is noted that these two models are not necessarily mutually exclusive and could apply to different target genes in different tissues.

Several groups have proposed that the induction of *Nab2 *constitutes a negative feedback loop in which EGR activators induce expression of their own corepressor [2,26,39,53]. Our studies refine this model by showing that other neuregulin-regulated pathways direct *Nab2 *expression. In addition, recent publications have shown that Egr2 is required for certain gene repression mechanisms during myelination [11,12]. Therefore, we suggest that the induction of NAB proteins does not merely constitute a negative feedback loop, but rather, that co-induction of Egr2 and NAB proteins by axon-dependent signals (e.g. neuregulin/erbB) is required to form an Egr2/NAB complex that actively represses transcription of specific genes during peripheral nerve myelination (Figure 8). These results are consistent with our demonstration of direct repression of the *Rad *gene by an Egr2/NAB complex in Schwann cells [6,22], as well as recent publications implicating repression by the Egr2/NAB complex in macrophages and cardiomyocytes [23,63].

Our studies do not rule out the possibility that other EGR family members and/or other zinc finger proteins may partially compensate in maintaining *Nab2 *levels in the absence of Egr2. Indeed, previous work has shown that Egr1 and Egr2/Krox20 are coexpressed in Schwann cells at the onset of myelination (P1). In contrast by one month of age, Egr2 is exclusively expressed in myelinating Schwann cells, whereas Egr1 is confined to nonmyelinating Schwann cells [64]. *Egr1 *is induced ~2-fold in sciatic nerve of mice homozygous for a hypomorphic *Egr2 *allele [11], and our analysis indicates that *Egr1 *mRNA is induced about 1.5-fold in the complete absence of Egr2 (data not shown). Importantly, compensation by other Egr family members is not sufficient to maintain Nab1 levels despite the presence of several Egr1 binding sites, and fails to maintain expression of other Egr2 target genes like myelin protein zero, which is reduced up to 50-fold in the *Krox20/Egr2 *knockout [10-14], suggesting that non-EGR factors contribute significantly to regulation of Nab2 expression. Interestingly, regulation of NAB expression by Egr2 is consistent with previous observations that *Nab1 *and *Nab2 *are co-regulated with *Egr2 *after nerve crush injury [22,36]. However, the decrease in *Nab2 *expression following nerve injury would be consistent not only with regulation by Egr2, but also by loss of neuregulin-dependent (i.e. axon-dependent) induction of ETS factors, analogous to regulation of ETS factors by neuregulin signaling observed in other systems [44-46].

The involvement of ETS transcription factors in *Nab2 *regulation resolves several puzzling observations regarding *Nab1 *and *Nab2 *activation. First, *Nab2 *expression is more responsive than *Nab1 *to several stimuli including serum, neuregulin and NGF [2,22], even though the *Nab1 *promoter contains more EGR binding sites than *Nab2*. Second, *Nab2 *expression is already fairly elevated by E17 in profiling studies of peripheral nerve myelination, even though *Egr2 *levels do not peak until P10 [49]. Finally, a recent analysis of hematopoietic development showed that expression of the ETS factor, *PU.1*, induced expression of both *Egr2 *and *Nab2*, and the induction of *Nab2 *was shown to be Egr2-independent [23]. Our analysis of the *Nab2 *promoter provides a mechanism for Egr2-independent activation of *Nab2 *by ETS proteins.

## Conclusion

Our results demonstrate that Egr2 is directly involved in regulation of both NAB1 and NAB2 corepressors, indicating that axon-dependent induction of NAB expression is partially dependent on Egr2. However, the maintained expression of *Nab2 *in Egr2 deficient mice suggested that other factors could compensate for lack of Egr2. Transfection experiments and chromatin immunoprecipitation assays indicated that Ets2 and Etv1 specifically activate the *Nab2 *promoter, but not *Nab1*, which provides an explanation for the differential regulation of NAB corepressors in several developmental systems. Finally, these results suggest that NAB corepressors are not simply feedback inhibitors, but that co-induction of Egr2 with NAB corepressors is critically involved in forming a transcriptional complex required for gene regulation events.

## Methods

### Promoter Analysis

Putative Egr2 binding sites were identified by comparison to the previously defined consensus Egr2 binding site [38], and sequence analysis for potential binding sites was performed using the rVISTA program [65]. Footprinting was carried out by incubating 200 ng of purified, FAM-labeled promoter fragments in the presence or absence of recombinant Egr2 protein, and 500 ng of a non-specific 20 bp oligonucleotide in binding buffer (20% glycerol, 20 mM Tris 7.5, 50 mM KCl, 5 mM MgCl_2_, 0.01 mM ZnCl_2_, 1 mM DTT, and 0.2 mM PMSF) in a volume of 10 μl. One microliter of 1000 U/ml DNAse I (Promega) was added for 1 min at room temperature before adding 90 μl of 0.5% SDS, 100 mM NaCl, and 10 mM EDTA. The resulting fragments were resolved on an ABI Prism 377 Sequencer. Recombinant Egr2 was made by fusing the mouse *Egr2 *sequence with the 6 × His tag in pET30a (Novagen), and purifying the protein from bacteria using Ni-NTA agarose (Qiagen) according to the manufacturer's protocol.

### Plasmids

A 370 bp NotI/PvuII fragment of the mouse *Nab1 *promoter (-250 to +120 relative to the transcription start site) and a 1000 bp KpnI/SmaI fragment of the mouse *Nab2 *promoter (-850 to +150) were fused to the luciferase gene in the pGL3 vector (Promega). The two upstream Egr2 binding sites in the *Nab2 *promoter were destroyed by replacing C residues at positions 2 and 8 with G. The Etv1 (ER81) expression plasmid was provided by Ralf Janknecht. The expression construct for Egr2 has been previously described [4]. A mouse Ets2 expression plasmid was provided by Barbara Graves [66].

### Cell culture conditions and transfection assays

JEG3 cells (a trophoblast-derived cell line) were transfected as described [43,54]. The average luciferase activity of each sample was normalized to the β-galactosidase activity from the transfected lacZ reporter. Unless otherwise indicated, means and standard deviations of two separate transfections are shown. The S16Y rat Schwann cell line [50] was maintained in DMEM (Dulbecco's Modified Eagle's Medium) supplemented with 10% bovine growth serum (Hyclone). Primary rat Schwann cells were cultured as described [43].

### Expression analysis

Sciatic nerve cDNA from pooled (or individual) *Egr2 *wild type, heterozygous and homozygous littermates at day P7 was provided by Lawrence Wrabetz and analyzed as described [14,41,43]. Quantitative RT-PCR was performed by monitoring in real-time the increase in fluorescence of the SYBR-GREEN dye [67] using the TaqMan 7000 Sequence Detection System (Applied Biosystems). Relative amounts of each gene between samples were determined using the Comparative Ct method [68] and normalized to the relative levels of 18S rRNA.

### Chromatin immunoprecipitation (ChIP) assays

All experiments were performed in strict accordance with experimental protocols approved by the University of Wisconsin, School of Veterinary Medicine. ChIP analysis of myelinating rat sciatic nerve at postnatal day 10 was performed as described [14,41], by mincing pooled sciatic nerves from 8–13 rat pups in 1% formaldehyde for 25 min. For neuregulin stimulation, primary rat Schwann cells were washed once in serum-free media and cultured in N2 medium for 24 hours [36]. The cells were treated with 20 ng/ml neuregulin-1 β isoform (heregulin-β 1, R&D Systems) for 2 h prior to cross-linking cells with 1% formaldehyde. Cross-linked cells were sonicated in a Biorupter sonicator (Diagenode) for 20 minutes.

For each immunoprecipitation, 300 μg of sonicated chromatin (as determined by Bio-Rad protein assay) from either nerves or cells was combined with Egr2 antibody (Covance) or rabbit IgG control antibody (Upstate) overnight at 4°C. 10% of this amount was saved as input. Chromatin-antibody complexes were collected with 30 μl protein G sepharose (pre-blocked with herring sperm DNA and Bovine serum albumin) for 1 hour at 4°C. Beads were washed with low salt, high salt and lithium chloride buffers as described [14,41]. Crosslinks were reversed for 5 h at 65°C in buffer containing 1% SDS, 0.1 M NaHCO3 and 200 mM NaCl and DNA was purified using QIAquick PCR purification kit (Qiagen). Quantitative PCR was carried out on the purified samples in duplicate with primers specific to regions of interest. Percentage recovery relative to input DNA was determined using the Comparative Ct method [68]. ChIP analysis in the S16Y Schwann cell line [50] was performed similarly with an affinity purified Ets2 antibody [51].

Primer sets used for ChIP analysis were as follows: *IMG2a *[41],

Rat *Nab1*: F) GGCTTGGCAGGCAGCAT R) GTCGGAGGAGCAGGCTTCT

Rat *Nab2 *promoter #3 (used in Figures 2, 3, and 6): F) ATAGCTCGGCCTCGGTCAC

R) GGGACTCAAGAATCGGGCTC, and the following primer sets used in Figure 6

*Nab2 *#1: F) CGGACCCCTCTCCAACTTTT, R) GCCATGCCGTTAGTTTGAATG

*Nab2 *#2: F) GTACGTGCGCGTGTCTTTGTA, R) AGAGCGCAGTTTCACAACCAC

*Nab2 *#4: GGGCAGAGGCACAGACAGA, R) CCTCCCTCCACGTCTTCTCTC

### Immunoblotting analysis

Sciatic nerves were dissected from Sprague-Dawley rat pups at postnatal day 5 (P5) and day 15 (P15) and homogenized in buffer containing 300 mM NaCl, 50 mM Tris pH 7.5, 1% Triton X-100, 10 mM EDTA pH 8.0, 1 μg/mL of pepstatinA, 1 μg/mL Leupeptin and 0.1 mM phenylmethylsulfonyl fluoride. The lysates were then analyzed by immunoblotting with Ets2 [51], Egr2 (Covance) or ETV1/ER81 (Santa Cruz, CA) antibody as indicated in figures. The membranes were reprobed with β-Actin (JLA-20, Developmental Studies Hybridoma Bank at University of Iowa) for loading control. The membranes were probed with horseradish peroxidase-conjugated anti-rabbit or anti-mouse secondary antibody (Jackson Laboratories, Bar Harbor, ME, USA). Luminescence was detected with West pico or west dura chemiluminescence detection system (Pierce, Rockford, IL, USA).

## List of abbreviations

EGR (early growth response) ChIP (chromatin immunoprecipitation), IMG2a (immunoglobulin G 2a), CMT (Charot-Marie Tooth)

## Authors' contributions

RS performed transfections, immunoblotting and ChIP experiments and helped write the manuscript. SJ assisted in development of in vivo ChIP assays and contributed preliminary data. RW performed DNase I footprinting. SS and TE produced and purified anti-Ets2 sera for ChIP analysis. JS was responsible for experimental design and analysis and drafted this manuscript. All authors read and approved the final manuscript.
